# Alcoholism is the mental health issue that best predicts the mortality of individuals experiencing homelessness.

**DOI:** 10.1192/j.eurpsy.2024.401

**Published:** 2024-08-27

**Authors:** F. Calvo, C. Giralt, X. Solench-Arco, X. Carbonell, S. Font-Mayolas

**Affiliations:** ^1^Departament de Pedagogia, Institut de Recerca sobre Qualitat de Vida; ^2^ Institut Català de la Salut, Centre d’Atenció Primària Blanes 2; ^3^Universitat de Girona, Girona; ^4^FPCEE Blanquerna, Barcelona; ^5^Departament de Psicologia, Institut de Recerca sobre Qualitat de Vida, Girona, Spain

## Abstract

**Introduction:**

The mortality rate among individuals experiencing homelessness tends to be premature and is linked to mental disorders and chronic diseases. In Spain, there is a significant gap in the study of mortality among individuals in situations of residential exclusion with real clinical data.

**Objectives:**

This study aims to analyze mortality among individuals experiencing homelessness and its relationship with mental disorders and chronic diseases.

**Methods:**

An observational and prospective longitudinal study was conducted on a cohort of 855 homeless individuals in the province of Girona over a 15-year period. Sociodemographic variables, mental health conditions, chronic diseases, and infections were analyzed, employing descriptive and inferential analyses. A binary logistic regression model was created to establish explanatory relationships between mortality and associated variables.

**Results:**

Among the participants, 87.7% were males with an average age of 53.03 years. A majority of 62.8% were foreign-born, mainly from Africa and Europe. It was identified that 40.8% had mental disorders, with substance dependencies (41.3%) and other disorders (36.4%) being the most prevalent. A total of 30.6% presented chronic diseases, notably hypertension (12.8%) and type 2 diabetes (10.9%). Furthermore, 22.3% had infections, with hepatitis C virus (8.7%) and HIV (4.7%) being the most common. During the follow-up period, 81 individuals (16.4%) passed away, with causes such as cancer (25%), suicide (21.7%), and heart conditions (11.7%).

The regression analysis demonstrated that age (OR = 0.915; 95% CI 0.884-0.947), alcohol addiction (OR = 2.354; 95% CI 1.486-3.731), and being born in Spain (OR = 2.906; 95% CI 1.594-5.299) were significantly associated with mortality in the homeless population.

**Conclusions:**

This study highlights the high prevalence of mental disorders, chronic diseases, and infections among individuals experiencing homelessness. Mortality was associated with factors such as age, alcohol addiction, and place of birth. These findings underscore the importance of developing interventions aimed at enhancing the health and care of individuals experiencing homelessness, particularly within the immigrant population.

**Disclosure of Interest:**

None Declared

